# Specific immediate early gene expression induced by high doses of salicylate in the cochlear nucleus and inferior colliculus of the rat^☆^

**DOI:** 10.1016/j.bjorl.2016.02.011

**Published:** 2016-04-25

**Authors:** Paula Santos, Lilian Eslaine Costa Mendes da Silva, Ricardo Maurício Leão

**Affiliations:** a Universidade de São Paulo (USP), Faculdade de Medicina Ribeirão Preto (FMRP), Departamento de Oftalmologia e Otorrinolaringologia e Cirurgia de Cabeça e Pescoço, Ribeirão Preto, SP, Brazil; b Universidade de São Paulo (USP), Faculdade de Medicina Ribeirão Preto (FMRP), Departamento de Fisiologia, Ribeirão Preto, SP, Brazil

**Keywords:** Salicylate, Tinnitus, Cochlear nucleus, Inferior colliculus, Salicilato, Zumbido, Núcleo coclear, Colículo inferior

## Abstract

**Introduction:**

Salicylate at high doses induces tinnitus in humans and experimental animals. However, the mechanisms and loci of action of salicylate in inducing tinnitus are still not well known. The expression of Immediate Early Genes (IEG) is traditionally associated with long-term neuronal modifications but it is still not clear how and where IEGs are activated in animal models of tinnitus.

**Objectives:**

Here we investigated the expression of c-fos and Egr-1, two IEGs, in the Dorsal Cochlear Nucleus (DCN), the Inferior Colliculus (IC), and the Posterior Ventral Cochlear Nucleus (pVCN) of rats.

**Methods:**

Rats were treated with doses known to induce tinnitus in rats (300 mg/kg i.p. daily, for 3 days), and c-fos and Egr-1 protein expressions were analyzed using western blot and immunocytochemistry.

**Results:**

After administration of salicylate, c-fos protein expression increased significantly in the DCN, pVCN and IC when assayed by western blot. Immunohistochemistry staining showed a more intense labeling of c-fos in the DCN, pVCN and IC and a significant increase in c-fos positive nuclei in the pVCN and IC. We did not detect increased Egr-1 expression in any of these areas.

**Conclusion:**

Our data show that a high dose of salicylate activates neurons in the DCN, pVCN and IC. The expression of these genes by high doses of salicylate strongly suggests that plastic changes in these areas are involved in the genesis of tinnitus.

## Resumo

**Introdução:**

Salicilato em doses elevadas induz zumbido nos seres humanos e em animais experimentais. No entanto, os mecanismos e *loci* de ação do salicilato na indução de zumbido ainda não são bem conhecidos. A expressão dos genes precoces imediatos (GPIs) está tradicionalmente associada a alterações neuronais em longo prazo, mas ainda não está claro como e onde os GPIs são ativados em modelos animais de zumbido.

**Objetivos:**

No presente estudo investigamos a expressão de c-fos e Egr-1, dois GPIs, no núcleo coclear dorsal (NCD), colículo inferior (CI) e núcleo coclear ventral posterior (NCVp) de ratos.

**Métodos:**

Os ratos foram tratados com doses que, conhecidamente, induzem zumbido em ratos (300 mg/kg IP/dia, por três dias) e as expressões das proteínas c-fos e Egr-1 foram analisadas por meio de *Western blot* e imunoistoquímica.

**Resultados:**

Após a administração de salicilato, a expressão da proteína c-fos aumentou significativamente no NCD, NCVp e CI, quando analisados por *Western blot*. A coloração imunoistoquímica mostrou uma marcação mais intensa de c-fos no NCD, NCVp e CI e um aumento significativo de núcleos positivos de c-fos no NCVp e CI. Não detectamos aumento da expressão de Egr-1 em nenhuma dessas áreas.

**Conclusão:**

Nossos dados mostram que uma dose alta de salicilato ativa neurônios no NCD, NCVp e CI. A expressão desses genes por doses altas de salicilato sugere que as alterações plásticas nessas áreas estão envolvidas na gênese do zumbido.

## Introduction

Tinnitus is a phantom sound sensation that can be the consequence of several factors including acoustic trauma, drugs, temporal mandibular disorders or deafness.^1^ The mechanisms of tinnitus induction and maintenance are still a matter of debate, especially since tinnitus can result from several different conditions. High doses of salicylate induce tinnitus in humans, and at doses of 150–400 mg/kg it induces behavioral symptoms of tinnitus in experimental animals in less than 24 h; this effect subsides within 72 h after treatment interruption.^2^

Despite the common use of salicylate as a tinnitus-inducing agent, its mechanisms and loci of action are still obscure. In vivo imaging experiments in rats have shown that high doses of salicylate induces hyperactivity in specific auditory areas, including the Inferior Colliculus (IC), the Dorsal Cochlear Nucleus (DCN) and the Auditory Cortex (AC), but not the Ventral Cochlear Nucleus (VCN).^3^, ^4^ On the other hand, studies of expression of the immediate early gene c-fos showed less consistent results. A single dose of salicylate (350 mg/kg) increased c-fos expression only in the AC of gerbils.^5^ A previous study did not show c-fos expression in the auditory brainstem after a single dose of salicylate, but only in non-auditory areas such as the locus coeruleus and periaqueductal gray area.^6^ Another study observed a decrease of c-fos expression in the IC of gerbils.^7^ Another study using chronic treatment with salicylate (250 mg/mL) showed increased c-fos expression only in the IC and not in the DCN.^8^ Most of these data are inconsistent with the observations that tinnitus induced by salicylate activates extralemniscal auditory pathways, especially the DCN.^2^, ^3^

The expression of Immediate Early Genes (IEGs) is considered a marker of increased brain activity in response to diverse stimuli. These genes are transcription factors that trigger the expression of other genes responsible for long-term changes in neurons. The expression of the IEG c-fos is a commonly used marker of neuronal activity and it is quickly upregulated after neuronal stimulation.^9^, ^10^, ^11^, ^12^ The IEG Egr-1 is activated in response to neuronal calcium influx and promotes functional and structural changes in neurons, including in the auditory system.^13^, ^14^

In this study, we aimed to investigate the activation of c-fos and Egr-1 in the DCN and IC in auditory pathways of rats subjected to a protocol of salicylate administration, which is effective in inducing tinnitus in rats (3 daily doses of 300 mg/kg).^3^ Due to its proximity to the DCN we also studied the expression of these genes in the posterior division of the Ventral Cochlear Nucleus (pVCN).

## Methods

### Animals and drug treatment

All experimental procedures performed on animals were approved by the institution's Animal Care and Use Committee (protocol n° 011/2013) and followed the guidelines and recommendations of the National Institutes of Health on animal care. Experiments were performed on male Wistar rats weighing 60–65 g. Rats were group-housed four to five per cage and kept under a 12 hour light/dark cycle with food and water ad libitum.

Animals were injected i.p. with 300 mg/kg sodium salicylate (Sigma) (10 μL/g), dissolved in saline, for three consecutive days with a 24 hour interval between each dose. The animals were then anesthetized with isoflurane and killed by decapitation on the third day, 3 h after drug administration. Control groups were administered saline following the same protocol. This protocol of salicylate administration has been demonstrated to be effective in inducing tinnitus in rats.^3^

### Detection of Egr-1 and c-fos by western blotting

Forty animals were used for these experiments: 20 were naïve animals and 20 were submitted to salicylate treatment. Slides with fresh sections (90 μm) of the brainstem containing the pVCN, DCN and IC were obtained in a cryostat. To obtain a tissue punch of the desired area we used a metal cylinder with a pistil of 0.5 mm diameter. Due to their small size and contiguous location, the DCN and pVCN were extracted in the same punch. One punch was obtained from each of four animals and they were pooled to increase protein yield. The tissue was homogenized in a lysis buffer containing 137 mM NaCl, 20 mM Tris, 1% Igepal CA-630, 10% glycerol, 2 mM sodium orthovanadate, 1% sodium dodecyl sulphate, 50 mM sodium fluoride, 2 mM EDTA, and 10% protease inhibitor cocktail at pH 7.4. Tissue homogenates were centrifuged at 15,000 rpm for 10 min at 4 °C. Protein concentration in tissues homogenates was determined using a modified Lowry assay (DC Protein Assay, Bio-Rad). Aliquots containing 90 μg protein were dissolved in loading buffer and heated at 95 °C for 5 min, and the proteins separated by 7.5% Tris–glycine SDS-PAGE (GE Healthcare-Bioscience) and transferred to PVDF membranes (Amersham Biosciences).

Immunoblots were blocked with albumin 5% and incubated with primary antibodies at 4 °C. Primary antibodies included antibodies against c-fos and Egr-1 (1:1000; Santa Cruz Biotechnology). After incubation, membrane was washed and incubated with secondary antibody (1:10,000; ECL anti-Rabbit IgG; GE Healthcare) for 1 h at room temperature. After final washes, labeled proteins were detected by chemiluminescence (RPN2132; GE Healthcare). For stripping and reprobing, the membrane was submerged in stripping buffer (100 mM 20-mercaptoethanol, 2% sodium dodecyl sulphate, 62.5 mM Tris–HCl, pH 6.8) at 50 °C, for 8 min, washed for 90 min under tap water, rehydrated with methanol and washed with TBS-T before blocking and reprobed with primary antibody against GAPDH (1:5000; Abcam). Western blots were photographed and quantified with image analysis (Molecular Imaging Systems).

### c-fos Immunohistochemistry

Ten rats were used, 5 naïve and 5 submitted to salicylate treatment. Rats were perfused transcardially with 0.9% NaCl followed by 4% paraformaldehyde in 0.1 M phosphate buffer (pH 7.4). The brain was removed, postfixed in the same solution for 1 h, and cryoprotected with 30% sucrose in 0.1 M phosphate buffer for 2 days at 4 °C. The brains were individually sectioned (50 μm) in the transverse plane using a cryostat.

The sections were washed in PBS (0.01 M, pH 7.4) and incubated for 30 min in PBS containing 1% hydrogen peroxide to inactivate endogenous peroxidases. After several rinses in PBS for 30 min, the sections were placed in 5% normal goat serum (Vector) for 1 h and incubated for 48 h at 4 °C with primary anti c-fos antibody generated in rabbits (1:100; Santa Cruz). After rinsed in PBS, the sections were incubated for 1.5 h at room temperature with biotinylated goat anti-rabbit IgG (1:150; Vector); then, subsequently, washed in PBS and placed for 1.5 h in avidin–biotin peroxidase complex (Vectastain, Vector). The immunolabeling was revealed by 5–10 min of incubation with 0.05% 3,3′-diaminobenzidine tetrachloride and 0.1% hydrogen peroxide. The sections were mounted on gelatin-coated slides, dehydrated, cleared with xylene and coverslipped with Entellan^®^.

### Data analysis

Western blot data are presented as the ratio of c-fos and GAPDH signal intensity. Stained nuclei in ICC sections were counted manually. Eleven sections containing the IC, 9 containing the DCN and 6 containing the pVCN were counted for each animal. All data are expressed as mean ± SD. Statistical analysis was carried out using Student's *t*-test. *p* < 0.05 was considered statistically significant.

## Results

**Figure 1:**
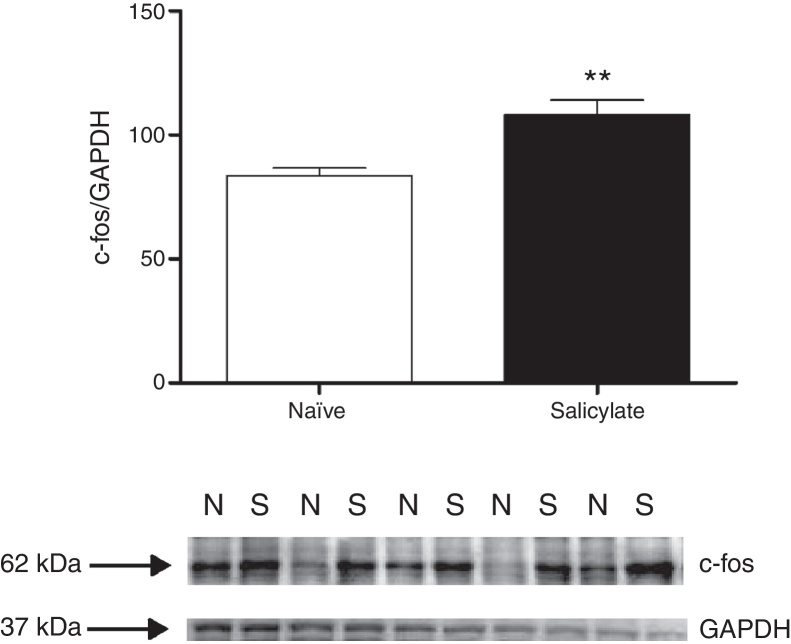
c-fos expression in the CN. Top. quantification of c-fos expression. The c-fos signal intensity was normalized to the GAPDH signal intensity (**p = 0.0058; *n* = 5 for each group containing material from 4 animals). Western blots showing the immunoreactivity of c-fos and GAPDH in punches containing the CN (N, naïve; S, salicylate).

**Figure 2:**
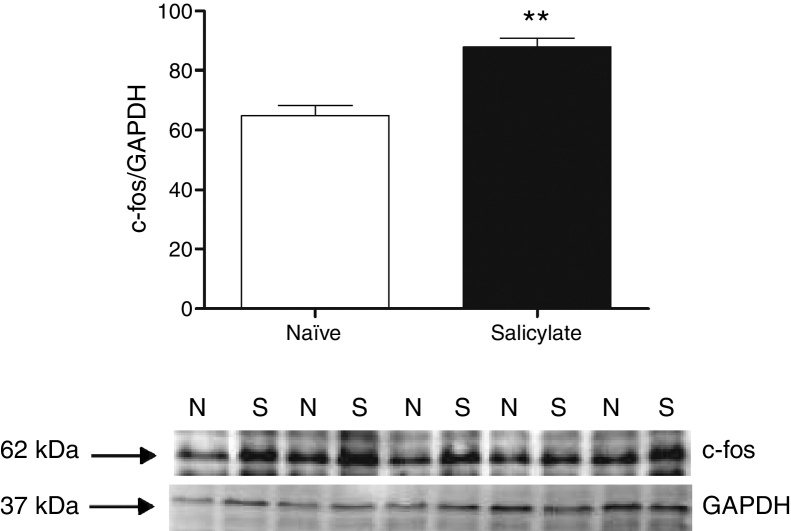
c-fos expression in the IC. Top quantification of c-fos expression. The c-fos signal intensity was normalized to the GAPDH signal intensity (**p = 0.0012; *n* = 5 for each group containing material from 4 animals). Western blots showing the immunoreactivity of c-fos and GAPDH in punches containing the IC (N, naïve; S, salicylate).

**Figure 3:**
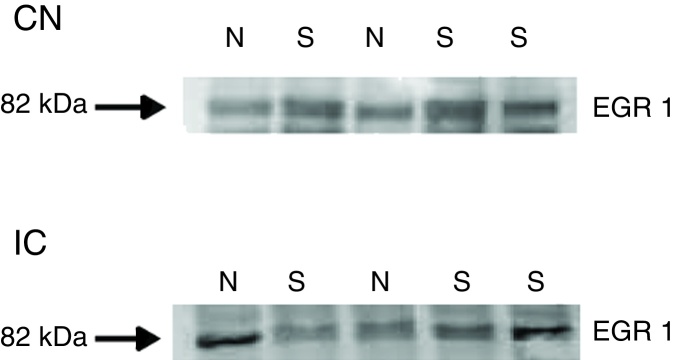
Western blots showing the immunoreactivity of Egr-1 in punches containing the DCN/pVCN and IC from naïve rats and after sodium salicylate treatment (N, naïve; S, salicylate).

After 3 days of salicylate treatment (300 mg/kg) we removed punches of brainstem containing the IC and both the DCN and pVCN for western blot analysis of c-fos protein expression. In both areas c-fos was significantly increased after salicylate treatment (Figure 1, Figure 2). In contrast, increased Egr-1 protein expression was not detected after salicylate treatment in these regions (Fig. 3).

**Figure 4:**
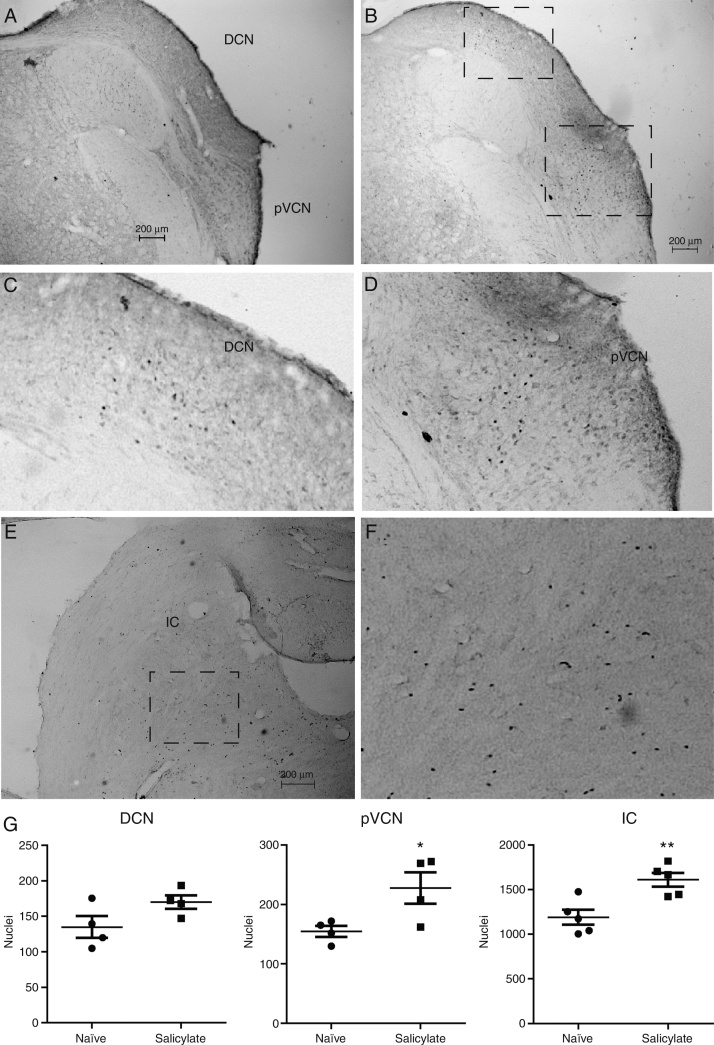
Detection of c-fos by immunocytochemistry in the DCN and IC. (A) Section containing the DCN and posterior Ventral Cochlear Nucleus (pVCN) from a naïve animal. (B) Section containing the DCN and posterior Ventral Cochlear Nucleus (pVCN) from an animal treated with salicylate. (C) Detail of the c-fos expressing nuclei in the DCN from the square area shown in (B). (D) Detail of the c-fos expressing nuclei in the pVCN from the square area shown in (B). (E) Section containing the IC from an animal treated with salicylate. (F) Detail of the c-fos expressing nuclei in the IC from the square area shown in (E). (G) Quantification of the expression of c-fos in the DCN, pVCN and IC [*p = 0.04; **p = 0.006; *n* = 4 (DCN, pVCN) and 5 (IC)].

Because the location of the c-fos protein cannot be identified by Western Blot, we performed c-fos immunocytochemistry in sections containing the DCN, pVCN and the IC. We detected c-fos in sections of the DCN, pVCN and IC, as shown in Fig. 4. In sections from naïve animals faint nuclei staining was observed in all 3 regions (Fig. 4A). In sections from salicylate treated animals we observed more intense labeled nuclei in DCN, pVCN and IC (Fig. 4B). In the DCN this intensely c-fos labeling was observed in the more internal layers, the deep layer and possibly the fusiform layer (Fig. 4B and C), and it was observed mostly in the medial/dorsal section of the DCN. We did not observe labeling in the molecular layer. We also observed increased c-fos labeling in the pVCN adjacent to the DCN (Fig. 4B and D). In the IC we observed an increase in the immunoreactivity mostly in the central part, as can be seen in Fig. 4E and F. No labeling was observed in the superior olive (not shown).

Quantitative analysis of nuclei expressing c-fos showed a significant increase of c-fos positive nuclei in the pVCN (naïve: 155 ± 9.2 nuclei; salicylate: 228 ± 26.4 nuclei; *p* = 0.04, *n* = 4) and in the IC (naïve: 1190 ± 86 nuclei; salicylate: 1611 ± 79 nuclei; *p* = 0.0061; *n* = 5), but not in the DCN (naïve: 135 ± 15 nuclei; salicylate: 170 ± 9.5 nuclei; *p* = 0.1; *n* = 4), as can be seen in Fig. 4G.

## Discussion

Our data show that 3 days of treatment with high doses of salicylate increase the expression of the products of the IEG c-fos, but not of Egr-1, in pVCN, DCN and IC of rats. Although we observed increased c-fos protein levels and a more intense nuclei labeling in the IC, DCN and pVCN after salicylate, the number of stained nuclei was significantly increased only in the pVCN and IC and not in the DCN. This data is in accordance with the observed hyperactivity of the DCN and IC in rats submitted to a similar protocol of salicylate treatment^3^, ^15^, ^16^ and in other animal models of tinnitus.^17^, ^18^ On the other hand, we found a significant increase in c-fos expression in the adjacent pVCN, a region not usually related to tinnitus induction.^3^ This increased expression of c-fos in DCN, pVCN and IC suggests that the expression of these IEGs promotes the expression of proteins that can change the physiological and morphological properties of the neurons of these regions, which could be a substrate for tinnitus induction.

Others^4^, ^5^, ^6^, ^7^ studied c-fos expression after acute salicylate treatment using immunocytochemistry. Those studies found that c-fos expression was not consistently increased in the DCN and IC. Our results on the other hand show a clearly increased expression of c-fos positive nuclei in the IC and pVCN. We believe these discrepancies can be attributed to the fact that we used younger animals and that those studies used a single dose of sodium salicylate, instead of the 3 daily doses used by this study. Consistent with this hypothesis, a study using 6 days of treatment with salicylate also observed a significant increase of c-fos positive nuclei in the IC, but not in the DCN.^8^

In experimental animals salicylate treatment increases the firing of neurons in both DCN and IC.^15^, ^16^ The mechanisms of this effect are still unknown and could reflect a direct effect of salicylate on the neuronal excitability of these neurons or their incoming synapses, or a downstream effect of salicylate-induced gene expression. Contrary to a direct excitatory effect of salicylate, it has been shown that direct application of salicylate on DCN neurons in brain slices decreases the spontaneous and evoked firing of DCN principal neurons.^19^, ^20^ On the other hand, direct application of salicylate increases the firing of most IC neurons in vitro.^21^ Salicylate could also enhance the excitability of DCN and IC neurons by decreasing their glycinergic inhibitory transmission.^22^ However, glycinergic currents in DCN fusiform neurons were not affected by acute applications of millimolar concentrations of salicylate.^20^ Salicylate can also inhibit GABAergic receptors^23^ and enhance NMDA currents^24^ which could potentially disturb the excitation-inhibition balance. Because most DCN neurons fire spontaneously,^25^ a firing increase produced by salicylate might not increase significantly the number of c-fos expressing neurons, which could explain the non-significant effect of salicylate in the number of c-fos labeled nuclei in the DCN. On the other hand, our data showed a significant increase in c-fos expressing nuclei expression in the pVCN, suggesting that salicylate is activating its neurons, and that this region could be relevant for the perception of tinnitus.

Both c-fos and Egr-1 are expressed in the auditory pathways in response to sound and intracochlear stimulation.^10^, ^12^, ^26^, ^27^ Interestingly, in the DCN c-fos expression elicited by intracochlear stimulation was most observed in glycinergic interneurons in the molecular layer.^27^ This finding is intriguing because these interneurons do not receive input from the primary auditory pathway, but from the parallel fibers, which convey mostly somatosensory information. We only observed c-fos labeling near or in the deep layer of the DCN in both control and salicylate conditions, suggesting that c-fos is expressed mainly by neurons computing the auditory information. On the other hand, we did not find expression of Egr-1, a IEG that is linked to the formation of long-term plasticity^13^ after salicylate treatment, suggesting that salicylate does not trigger the expression of genes related to long-term synaptic plasticity. Interestingly, a previous study found a decrease in Egr-1 gene expression after salicylate treatment in the IC.^28^

There are similarities of tinnitus with neuropathic pain,^29^, ^30^ suggesting that they could share similar molecular mechanisms. For instance, patients with tinnitus often present with hyperacusis, and patients with chronic pain, hyperalgesia, both of which are enhanced reactions to normal stimuli intensities. Like tinnitus,^31^ chronic pain is believed to be a result of long-term plastic changes in sensory neurons and synapses.^32^ The IEG c-fos is also activated in the spinal cord in models of chronic pain and inflammation.^33^, ^34^ Their expression is postulated to be the first signal of long-term changes in these neurons which will lead to chronic pain. Thus, it is likely that c-fos is activating similar cascades of gene expression in tinnitus and chronic pain, leading to changes that would underlie the symptomatology of these conditions.

## Conclusion

Our data shows that tinnitus-inducing doses of salicylate^3^ increase c-fos, but not Egr-1, expression in auditory areas of the auditory brainstem, the dorsal cochlear nucleus, the posteroventral cochlear nucleus and inferior colliculus of the rat. Thus, we postulate that c-fos is activating cascades of gene expression in these areas leading to changes that could underlie the symptomatology of tinnitus.

## Conflicts of interest

The authors declare no conflicts of interest.
